# “Missed” Diagnosis of Mycosis Fungoides: A Case Report

**DOI:** 10.1002/ccr3.70172

**Published:** 2025-02-05

**Authors:** Mahesh Mathur, Neha Thakur, Sandhya Regmi, Supriya Paudel, Sambidha Karki, Nabita Bhattarai

**Affiliations:** ^1^ Department of Dermatology College of Medical Sciences Teaching Hospital Bharatpur Nepal

**Keywords:** clinical dermatology, cutaneous T‐cell lymphoma, histopathology, mycosis fungoides, non‐Hodgkin lymphoma

## Abstract

Mycosis fungoides (MF) is the most common subtype of primary cutaneous T‐cell lymphoma characterized by malignant proliferation of T cells with epidermotropism in the skin. MF has an indolent course, presents as erythematous scaly patches or plaques, and may progress to generalized erythroderma, cutaneous tumors, or extracutaneous invasion. MF is often misdiagnosed at early stages due to nonspecific clinical findings. Patients with MF are at high risk for developing secondary malignancies, including hematological malignancies. We hereby report a case of MF misdiagnosed and associated with underlying diffuse large B‐cell lymphoma.

**Abbreviations**
CD: cluster of differentiation
MF: mycosis fungoides
R‐CHOP: rituximab‐ cyclophosphamide, doxorubicin, vincristine, prednisone
TNMB: tumor‐node‐metastasis‐blood

**Summary**

- Mycosis fungoides (MF), a subtype of primary cutaneous T‐cell lymphoma, is often misdiagnosed at early stages due to nonspecific clinical findings.
- Patients are at high risk for developing secondary malignancies.
- MF should be suspected in patients with underlying malignancies and patients with MF should be screened for secondary malignancies.

## 1 Introduction

Mycosis fungoides (MF), although uncommon, is the most common subtype of primary cutaneous T‐cell lymphoma that occurs in middle aged and elderly adults [1]. MF has an indolent course, presents as erythematous scaly patches or plaques, and may progress to generalized erythroderma or cutaneous tumors. MF is often misdiagnosed at early stages due to nonspecific clinical and pathological findings, and prognosis depends on the type and extent of skin involvement and extracutaneous invasion [2]. Patients with MF are at high risk for developing secondary malignancies, including hematological malignancies [3, 4]. We hereby report a case of MF misdiagnosed and associated with underlying diffuse large B‐cell lymphoma.

## 2 Case Presentation

**FIGURE 1:**
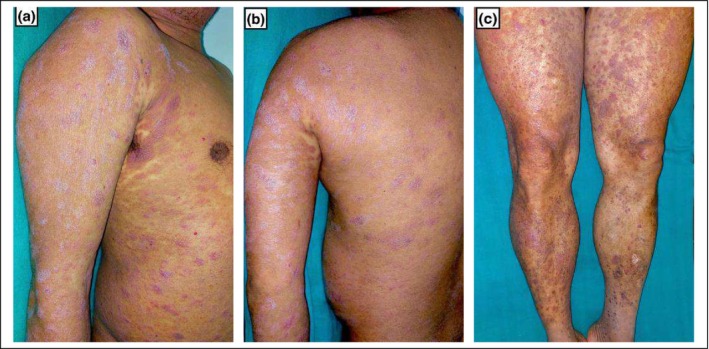
Multiple ill‐defined, scaly, erythematous‐violaceous patches and plaques over right arm and anterior trunk (a), left arm and posterior trunk (b) and bilateral thighs and legs (c).

A 50 year old man presented with multiple itchy, ill‐defined, scaly, erythematous‐violaceous patches, and plaques over generalized body surface for 7 years (Figure 1a–c). Initially, skin lesions appeared bilaterally over the thighs and buttocks, for which he visited a medical center and was prescribed antileprosy medication for 1 year. He was also treated with topical steroids on and off for chronic dermatitis with minimal improvement. He had an abdominal mass excised 2 years back, and histology reports were suggestive of diffuse large B‐cell lymphoma, so he received R‐CHOP regimen for six courses. Notably, the cutaneous lesions significantly improved while on chemotherapy. However, the skin lesions flared up after 6 months of stopping the chemotherapy.

## 3 Methods

**FIGURE 2:**
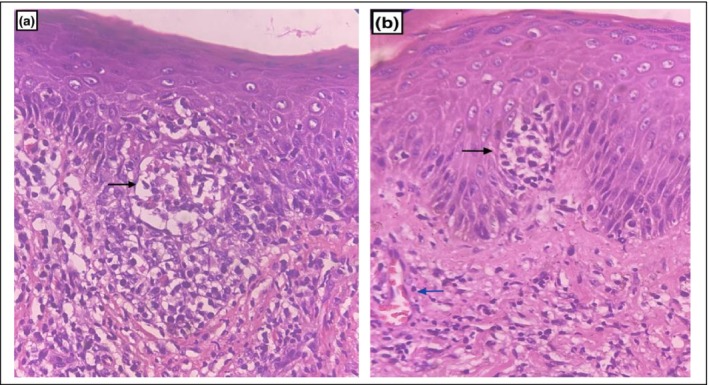
(a, b) Hematoxylin and eosin staining (40×) showed epidermotropism, Pautriner's microabcesses (black arrow) and perivascular lymphocytic infiltrates (blue arrow).

Routine blood investigations revealed normal findings. Skin biopsy showed epidermotropism, Pautrier's microabscesses, and perivascular lymphocytic infiltrates (Figure 2a,b). Immunohistochemistry revealed intraepidermal and perivascular atypical cells, which were immunopositive for CD2, CD3, CD4, CD5 with Ki‐67 proliferation index of 20% and negative for CD20. Bone marrow aspiration and bone marrow biopsy performed were normal. CT scan reports of the neck, chest, abdomen, and pelvis were normal. Based on clinical, histopathological, and immunohistochemistry findings, the patient was diagnosed as case of MF, a subtype of primary cutaneous T‐cell non‐Hodgkin lymphoma.

## 4 Results

As he was diagnosed in the early stage (stage 1B) according to the modified tumor‐node‐metastasis‐blood (TNMB) classification, was started on low‐dose methotrexate and is under regular follow‐up.

## 5 Discussion

MF is characterized by malignant proliferation of T cells with epidermotropism in the skin [1]. The classical MF has been defined as a three‐stage disease characterized by the progressive appearance of patches, plaques, and tumors; however, not all the patients progress through these three stages and can develop patches, plaques, or tumors as a presenting form of the disease. Various variants of MF described in the literature are hypopigmented, hyperpigmented, erythrodermic, ichthyosiform, papular, pustular, purpuric, solitary or unilesional, invisible, folliculotropic, syringotropic, anetodermic, verrucous, bullous, dyshidrotic, granulomatous, papillomatous, mycosis fungoides palmaris et plantaris, mycosis fungoides with eruptive infundibular cysts, pagetoid reticulosis or Woringer–Kolopp disease, poikilodermal, interstitial, granulomatous slack skin, and mycosis fungoides with large‐cell transformation [5]. The diagnosis of MF is often delayed by many years from the initial appearance of skin lesions due to its indolent course and diagnostic difficulties, as in our patient. In the early stages of disease, it can mimic common skin conditions such as chronic eczema, tinea corporis, tinea pedis, erythema multiforme, annular erythema, drug reaction, vitiligo, leprosy, pityriasis alba, verruca vulgaris, psoriasis, and atopic dermatitis [1, 6].

Predisposing factors that induce the development of MF are not fully understood, though it is hypothesized that genetic and environmental triggers, pharmacologic treatments, or other underlying cancers may contribute [3, 4]. Viral infections, genetic factors, and environmental exposures implicated in carcinogenesis might be the common mechanism linking the onset of MF with increased risk for a second malignancy [4]. Furthermore, altered T‐cell activation in MF might contribute to develop secondary lymphoid malignancy, as it is evident that a subset of highly immunosuppressive regulatory T cells emerging from the infiltrating malignant T cells increases the risk of severe infections and malignancy in MF [3, 4].

The most common malignancies seen in patients with MF are hematologic malignancies, including Hodgkin and non‐Hodgkin lymphomas, and solid tumor malignancies, including melanoma, lung, female breast, prostate, bladder, colon, pancreas, and kidney [7]. Our patient developed MF initially, which was underdiagnosed, and subsequently he developed diffuse large B‐cell lymphoma (DLBCL). The lesions of MF improved while the patient received chemotherapy for DLBCL and relapsed after stoppage of chemotherapy. Similar to our case, Rohan et al. [8] reported an 81‐year‐old woman with early‐stage MF who simultaneously progressed to tumor stage, large‐cell transformed (LCT) MF, and developed a primary DLBCL in a lymph node. Similarly, Miyatake et al. [3] reported two cases of MF concurring with diffuse large B‐cell lymphoma (B lymphoid lineage) and acute myeloid leukemia (myeloid lineage) in two otherwise healthy elderly patients. Miyagaki et al. [9] reported a 75‐year‐old man with MF associated with a composite recurrent HL and diffuse large B‐cell lymphoma.

MF is classified into four stages according to the TNMB classification based on the extent of cutaneous involvement, the presence of nodal or visceral involvement, and the presence of Sezary cells at the peripheral blood level. Diagnosis is difficult especially in the early stages, but it is made through clinical examination and is confirmed by a skin biopsy, immunohistochemistry, and staged appropriately. Histologically, early MF is characterized by epidermotropism, atypical lymphocytes with cerebriform nuclei, Pautrier's microabscesses, and basal alignment of neoplastic lymphocytes as seen in our case. In immunohistochemistry, MF cells are positive for CD2, CD3, CD4, CD5 and negative for CD20 as reported in our case [3, 5]. Management is based on disease stage and includes skin‐directed therapies, systemic therapies, and allogenic stem cell transplantation [1, 2]. Skin‐directed therapies are topical corticosteroids, topical chlormethine, topical retinoids, topical calcineurin inhibitors, imiquimod, ultraviolet phototherapy, photodynamic therapy, localized radiotherapy, and total skin electron beam therapy. Systemic therapies include oral retinoids, interferon‐α, histone deacetylase (HDAC) inhibitors, low‐dose methotrexate, chemotherapeutic agents (pegylated liposomal doxorubicin, gemcitabine, and chlorambucil), and targeted immunotherapy (alemtuzumab, mogamulizumab, and brentuximab vedotin) [1, 10].

## 6 Conclusion

MF is a clinical diagnosis; however, is easily misdiagnosed at an early stage due to its nonspecific clinical features and lack of awareness, warranting a high index of clinical suspicion. MF should be suspected in patients with underlying malignancies and patients with MF are at high risk for developing secondary malignancies, which should be screened for and vice versa.

## Author Contributions

**Mahesh Mathur:** conceptualization, formal analysis, resources, supervision, validation, visualization, writing – original draft. **Neha Thakur:** conceptualization, formal analysis, resources, supervision, validation, visualization, writing – original draft. **Sandhya Regmi:** conceptualization, formal analysis, resources, supervision, validation, visualization, writing – original draft. **Supriya Paudel:** formal analysis, resources, supervision, visualization, writing – original draft, writing – review and editing. **Sambidha Karki:** data curation, investigation, resources, visualization, writing – review and editing. **Nabita Bhattarai:** data curation, investigation, resources, visualization, writing – review and editing.

## Ethics Statement

Reviewed and approved by Institutional review board College of medical sciences (IRBCOMS).

## Consent

Written informed consent was obtained from the patient to publish this report in accordance with the journal’s patient consent policy.

## Conflicts of Interest

The authors declare no conflicts of interest.

## Data Availability

The data that support the findings of this study are available from the corresponding author upon reasonable request.
